# Understanding end-of-life doula care provision: reporting on the design of a bereavement survey to evaluate doula support

**DOI:** 10.1177/26323524241273489

**Published:** 2024-10-17

**Authors:** Kirsten Bashir, Emma Clare, Catherine Pestano, Esther Ramsey-Jones, Erica Borgstrom

**Affiliations:** The Open University, Milton Keynes, UK; End of Life Doula UK, Derby, UK; The Open University, Milton Keynes, UK; The Open University, Milton Keynes, UK; The Open University, Walton Hall, Milton Keynes MK7 6AA, UK

**Keywords:** doula, end-of-life care, end-of-life doula, evaluation, survey design

## Abstract

**Background:**

Delivery of consistent patient-centred care at end-of-life care continues to challenge healthcare providers and research continues to suggest that peoples’ needs are not being reliably met. Consequently, healthcare services are looking to innovate how support is provided, such as commissioning doulas to support dying people and those close to them.

**Objective:**

Within the United Kingdom, there is little existing research about peoples’ experience of receiving end-of-life doula support. This paper outlines the design of a survey for the family or friends of a person who received end-of-life doula support.

**Design:**

To evaluate the role of an end-of-life doula in supporting the dying person and those who care for them, we designed a post-bereavement survey as part of a wider evaluation strategy of doula services. Following multiple literature reviews and an iterative process of consulting with the professional organisation and previous service users, a questionnaire was developed to collect this data. This survey is hosted online, with paper copies available to widen accessibility.

**Conclusion:**

End-of-life doula support is a relatively new area of provision for dying people and those important to them, such as family and friends. It is even more innovative to have doula support commissioned as part of a locality’s healthcare service. There is a dire need for empirical research to understand the impact of this further. The process of researching the area and designing the evaluation survey for this service revealed the complexity of the role and the difficulty of capturing what was found to be helpful for the dying person and those around them.

## Background

It has been suggested that how we care for the dying is a barometer for the standard of how we care for all sick and vulnerable people,^1^ yet current evidence would suggest that peoples’ needs are not being consistently met.^2^ This disparity is evident even in countries, like England, which are world-renowned for their palliative and end-of-life (EoL) care. Owing to this disparity, various interventions and policies have been created to enhance the quality and accessibility of EoL care within England as well as considering the wider death system and social context in which people die.^3^ One area of increasing innovation in this field of practice is the use of EoL doulas, which is a relatively new concept and emerging practice within the United Kingdom. Doulas seek to provide support and accompaniment through the dying process. This article focuses on the design of a post-bereavement survey instrument to understand doula support, developed as part of a wider evaluation project about doula provision commissioned by an Integrated Care Board in England.

### Palliative and EoL care in England

To understand the innovation of doula support in England, it is useful to understand the wider context in which palliative and EoL care and support are provided. The United Kingdom is thought of as the origin of the modern hospice movement since the 1960s,^4^ supporting a holistic approach to dying and resisting overly medicalising death. Despite the legacy of hospice and associated palliative care, there is still notable room for improvement. For example, in 2008 the first EoL national strategy in England, *End of Life Care Strategy: Promoting high quality care for all adults at the end of life*,^1^ was published against the backdrop of concerns regarding the quality of EoL care and difficult headlines like this from *The Guardian* Newspaper in 2005; ‘60 people a week dying alone at home, says MP’.^5^ This strategy spurred the health and social system to address three core issues: (1) that people often do not die in their place of choice; (2) that more preparation was required to meet the larger numbers of dying people and (3) that not everyone was consistently receiving high-quality care.^1^ Following this strategy, there were a number of reports and national guidelines^6,7^; however, perhaps most significant has been the coming together of different organisations to form The National Palliative and End of Life Care Partnership in England, with its main goal to improve EoL care across the nation. The National Palliative and End of Life Care Partnership published a revised framework for action to cover up to 2026.^2^ The framework has six core ambitions aimed at improving palliative and EoL care for all: each person is seen as an individual; each person gets fair access to care; maximising comfort and well-being; care is coordinated; all staff are prepared to care and each community is prepared to help. What is key about this partnership and framework is the recognition of the role third-sector provision has in contributing significantly to good-quality EoL care and support. EoL doulas are one such third-sector service that may help to meet these ambitions at a local level.

### Understanding the role of EoL doulas

The word doula originates from Ancient Greek meaning ‘female slave’ and whilst the term is problematised and contested in linguistic and colonial terms,^8^ this article uses the term as it is in widespread use within this field and has clearly evolved from the original etymology. Today, the meaning of doula is used to refer to a trained person who provides skilled guidance for the benefit of others and who supports another person through a key health-related event.^9^ Doulas may also give support to the client’s partner, family or friends.^10^ Doulas have been described as the ‘bookends of life’, so as a birth doula is present at the start of life, an EoL doula is there at the EoL. The focus of an EoL doula is on providing support to a person and those who are important to them when diagnosed with a terminal illness.^11^

Internationally and nationally, EoL doulas have an emerging and potentially important role in EoL care,^12^ especially in the West.^13^ EoL doula practices have in some places been developing over the past few decades,^14^ yet significantly there is no definitive identity aligned to the EoL doula with inconsistencies in how the role is described and enacted in both academic literature but also through the voices of those working within these roles.^15^ The role of EoL doula has been described as ‘amicus mortis’ meaning a friend in death.^15^ Another author suggests that EoL doulas act as knowledgeable confidants, going with, seeing and being present with the dying person. She argues the EoL doula supports the dying person practically, emotionally and spiritually working with individuals or families and in partnership with health professionals in supporting people at the EoL providing person-centred care. Furthermore, they foster conversations about death and dying and support the bereaved.^16^ A central theme in the limited EoL doula literature describes the doula’s role as providing comfort and care at the EoL,^17^ and supporting the dying person and those important to them to find peace at the EoL.^18^ Yet, since the doula’s role is relatively new, compared to other more established professional’s role around the EoL, there is little empirical research about the impact of their support and/or how people experience receiving doula services. Most research to date has focused on the perspectives of doulas themselves,^9,15^ with the recent exception of an interview study with 10 bereaved relatives who used a doula.^19^

The impact of the doula is beyond the individual dying person. Research literature to date illustrates that EoL doulas may make an important contribution to community-based care.^9,12^ Some posit that EoL doulas can not only improve the quality of dying^12^ but also help address inequalities in health systems by being with people underserved by healthcare services.^20^ Crucially in the context of EoL systems in need of improvement and innovation, EoL doulas confer the possibility to redesign and challenge the status quo and culture of EoL care.^21^ Arguably with sensitive exploratory commissioning of services, new understandings may be gained about the needs of the dying person and those around them, which may be addressed by EoL doulas.

It is important to note, however, that just as the role of the EoL doula is evolving, especially in the United Kingdom, how their support is provided similarly varies without a standard model to date.^21,22^ Some EoL doulas offer their services voluntarily, whereas other doulas provide a paid-for service which is typically paid privately by the person they are accompanying.^21^ Previous research has noted that the lack of a consistent EoL business model means that there is variation in what each service offers.^15^ This has implications both for how the public understands the doula role and also for healthcare commissioners who may wish to employ doulas within EoL care.

End of Life Doula UK is a Community Interest Company that acts as a community of practice for doulas and mediates referrals from individuals and healthcare professionals to doulas across the country. It typically provides a private self-referral service with individual doulas determining if they offer their time voluntarily or for a fee. However, from 2022, End of Life Doula UK has been commissioned by the NHS West Yorkshire Integrated Care Board (covering the areas around Leeds, England) to provide a pilot for their EoL doula service to dying people and those supporting them (such as family, friends and neighbours). This is a novel arrangement piloting EoL doula services within the UK healthcare system, using National Health Service (NHS) funding and funnelling referrals for EoL doula services through NHS services, such as general practitioners and acute hospital discharge teams. This commissioning is an example of Integrated Care Boards innovating in how they meet their statutory duty to provide palliative and EoL care in accordance with the Health and Care Act, 2022.^23^

The pilot provides funding for approximately 1500 h of doula services and some administrative support. As part of entering into the commissioning arrangement, End of Life Doula UK and the NHS commissioners agreed on several outcome measures. These focused on the support individuals and those around them were to receive from the EoL doula, including support with advance care planning, reducing unwanted hospital admissions in the last weeks of life, and enabling family/carers to feel supported and less anxious about the death including if the dying person is supported to die within the home. These were areas the local commissioners wanted to improve and were in alignment with several aspects of the Ambitions for Palliative and End of Life Care national framework.^2^

## Evaluation study: Designing a post-bereavement tool

### Objective

To evaluate the pilot, End of Life Doula UK has collaborated with researchers at The Open University. This article describes the design of one of the tools for this evaluation – a post-bereavement survey. The focus of this article is on the design of this instrument since at the time of writing the evaluation is still underway. The overarching research question for the evaluation was ‘how are doula services being provided’, and for this element, the focus was on what are the bereaved person’s experiences and ratings of doula provision for themselves and the dying person.

As evidenced in the existing literature about doulas, much of the research about doulas is from the perspective of doulas; this evaluation sought to not only meet the needs of End of Life Doula UK but also develop research methods that may enable a wider range of perspectives to be sought. It was important that the survey captured people’s perspectives and voices as well as identifying the range of support provided by EoL doulas to the dying person and those around them. There is much to be understood about individual EoL doula practices framed within the local, national and international priorities and this pilot seeks to add to and inform this knowledge.

### Design

The doula services were commissioned as a pilot. The purpose of a pilot study is to gather information to help improve a project or to evaluate its viability.^24^ Thus, it was essential in this survey that the voice of those supported by the doulas was heard; since doulas support people at the EoL, End of Life Doula UK wanted to conduct an element of evaluation that was post-bereavement.

Research conducted using surveys allows researchers to examine a situation as they can describe the reality and depending upon their design, can provide data relating to occurrence, frequency, behaviours, trends, viewpoints, traits and experiences.^25^ Surveys can be designed from either an interpretivist or positivist paradigm,^24,25^ depending upon the researcher’s design and intended outcomes. Adopting an interpretivist perspective, in this survey we sought to collect both quantitative data providing numerical data against the outcome measures, and also qualitative data enabling participants’ views and experiences to be collected and evaluated. In design, this was a descriptive survey as it aimed to describe the situation.^25^ The reporting of this design conforms to an adapted version (excluding all references to results) of the *Questions to consider when preparing a report of findings from postal surveys* statement.^26^

To inform the design of the survey, the researchers met with End of Life Doula UK to understand their outcome measures, their motivations for evaluation, and what they viewed as key aspects of the doula support/provision. End of Life Doula UK was keen to understand how bereaved family/friends/carers experienced the support provided by the doula across a range of outcome measures. This helped shape the literature review (below) and appraisal of existing measures in terms of relevance for the doula service and latterly the question design. We followed a seven step process, informed by Kasunic’s 2005 work to facilitate the design process. This involved: (1) Identifying the objectives of the survey; (2) Identifying the target audience; (3) Designing the sampling strategy; (4) Designing and writing the questionnaire; (5) Conducting a pilot test of the questionnaire; (6) Circulating the questionnaire and finally; (7) Analysing the results and writing the results.^27^

### Literature to inform survey tool development

The literature available on EoL care is vast and diverse. In determining how to provide a meaningful evaluation of the service which would not only evaluate the service but also provide suggestions for improvement, it was important to consider two key elements. Firstly, how this service would sit in current healthcare provisions, and secondly, were there any existing valid and reliable EoL assessment tools that would support this evaluation?

In 2022, an initial literature search using the Boolean/Phrase ‘end of life doula’ was undertaken in the Cinahl database; it produced an initial result of 26 articles that had been published between 2011 and 2022. This was reduced to 11 when the results were refined to only include full-text literature and four duplicates were removed due to publishing errors. A further search was undertaken in Medline using the same search parameters and 21 articles were identified although 7 were duplicates from the Cinahl search. The majority of these articles were from Australia and examined the role of EoL doulas within the Australian healthcare setting and whilst the role was explored, it was from the perspective of the doula and not those receiving support.^9,15^ Furthermore, none of the articles evaluated the role or service provision of EoL doulas.

An additional literature search was undertaken using both the Cinahl database and Google Scholar to explore quality-of-life assessment tools at the EoL. This was conducted to see whether there were tools within the wider EoL care context that could be useful for evaluating doula practices. This search initially produced thousands of articles in Google Scholar and hundreds in the Cinahl database. Articles looked at defining quality of life and in many cases specific symptom management such as pain, fatigue and breathlessness. Since doulas do not address symptom management (as this is within the remit of palliative care professionals), such articles were excluded.

One significant document identified during the search was the *Assessment Tools for Palliative Care* (2016) produced by the Agency for Healthcare Research and Quality.^28^ It sought to provide a systematic integrated review to support palliative care assessment and practice. Whilst 150 assessment documents were considered, they found gaps or limited tools within four of their key domains: spiritual aspects of care; structure and processes of care; cultural aspects of care and legal and ethical aspects of care. Perhaps even more significant was the limited evidence of tools supporting the patient-reported experience. These tools whilst undeniably valuable in EoL care did not meet the needs of our intended survey. Thus, we needed to refine the search to identify more specific tools to support the design of this survey that would apply to doula services.

Further scoping was undertaken to look for tools that were designed to measure the ‘quality of death, dying and care completed after death’. Identified in this was Kupeli et al.’s^29^ systematic review examining the psychometric properties of these tools. Whilst they found that there was limited evidence on the available tools, they identified the Care of the Dying Evaluation (CODE™) and Care of the End of Life in Dementia tools as having the best psychometric properties among the tools that measured quality-of-life care at the EoL.^29,30^ In addition, within this scoping, a Network report into the quality assurance for care of the dying was also identified that had been published by the *Cheshire & Merseyside Strategic Clinical Network Group in 2015*.^31^ Within this report, the CODE^30^ questionnaire was used to listen to the voices of bereaved relatives to understand their perspective of the quality of care and support provided to people and their families in the last days of life thus potentially providing us with a tool that would support the development of the survey.

Mayland et al. argued that the existing *Quality of Death Index* demonstrated inconsistency in the international delivery of care for the dying.^30,32^ To address this variability and to improve care, they reasoned that there was a need to have a validated outcome measure to assess and evaluate the current quality of EoL care. They suggested that one method of evaluation was to utilise the viewpoints of the bereaved relatives to consider their perception as a proxy measure for the person who had died. A core international Care of the Dying (i-CODE) was developed to assess both patient care and family-carer support.^30^ CODE was subsequently tested in seven countries and was shown to have face and content validity, construct validity and internal consistency within an international context.^30,33^

Overall, the literature search identified few tools that assess the quality of dying and none that specifically address doula support. We shared insights from the literature review with End of Life Doula UK to help determine survey topics and questions. The following section outlines this in more detail.

### Details about the survey

To evaluate the role of the EoL doula in supporting the dying person and those who care for them, we designed a post-bereavement survey as part of a wider evaluation strategy. A questionnaire was developed to collect this data. The intended survey participants were adults who were ‘close to’ the dying person but also known to the doula, such as a family member, friend, neighbour or another informal carer. The survey was designed to be completed online or on paper depending on the participants’ preference.

Overall, the final survey had a mixture of closed and open questions, with 18 questions in total split between 5 parts. Topics covered emotional support provided by the doula to the dying person and those close to them, practical support provided by the doula, whether advance care planning support was provided and rating of this, how involved the person felt with decision-making, information provision by the doula, place of death and how doula support may have impacted the person’s (i.e. family member’s) confidence and well-being in terms of providing care around death. There are also two free-text boxes enabling feedback on doula services and any other information the respondent wants to share. The questionnaire included questions relating to quality of care, key performance indicators aligned with the commissioning arrangement and questions aligned with other quality of EoL questionnaires, in particular CODE. The question format and response options adapted from CODE were questions relating to emotional support, care in the last 2 days and place of death. Permission was gained from the authors of CODE to utilise and adapt appropriate CODE questions within the survey. The full questionnaire is available online (Supplemental Material).^34^

The survey was built within the Jisc Online Survey platform (manufactured by Jisc, Bristol, UK). This enabled a range of question types to be developed and permitted question routing capability, and accessibility features. It is also compliant with the General Data Protection Regulations (GDPR). The participant information sheet is built into the start of the survey and full consent is received when a person submits their responses. If someone does not click ‘finish’, none of their answers are saved, thus enabling people to ‘withdraw’ their data and consent during participation. Each participant is provided an anonymised identity code by the platform which streamlines the research processes. Immediately after completing the survey, the system offers them an option to download a copy of their responses.

### Tool review and validity

To ensure the robustness of the tool, we undertook several steps. Firstly, where possible, we used questions from similar tools that have already been validated. This was primarily in relation to the questions adapted from CODE, which are outlined above. Utilising questions and response options that had already been tested supported the validity of the tool. Secondly, prior to launching the questionnaire, a draft of the survey was reviewed by a person who had previously received EoL doula support and who was able to comment on both accessibility and appropriate timing of the survey send-out. This review tested the appropriateness of the questions, the language in the questionnaire, length and overall burden to complete. This peer review helped to ensure readability and sensitivity thus ensuring face validity.^35^ Thirdly, End of Life Doula UK also reviewed the questions to ensure that the wording, order and answer types met their objectives and would provide usable data. Following steps two and three, minor changes were made before producing a definitive version of the survey, which was then reviewed and approved by the university ethics committee. Given the relatively small number of possible participants, a pragmatic decision was made not to conduct more elaborate validity tests, especially ones that rely on larger sample sizes.^36^

### Recruitment process

Once built, the survey was completed and launched in April 2022. Potential participants include those who were close to someone supported by an EoL doula in Leeds and the surrounding area. Whilst providing services, the doula is asked to inform family/friends about the survey and the purpose of the survey. Only one bereaved contact per client is sent the survey; in social networks where there is more than one person close to the client, the doula nominates the person they had the most contact with. Approximately 3 months after the death, End of Life Doula UK (the organisation, not the individual doula who provided the service) will send out the survey links to participants and up to one reminder email or message. This saw the survey being shared from July 2022 which coincided with being after The Open University ethical approval was granted. If the doula is aware that a family/friend is unable to complete an online survey or a survey in English, they will inform End of Life Doula UK who will make arrangements to send paper/translated versions of the survey. Access to complete the survey is via direct link only (or an adapted version where required); the survey was not shared on social media, in publications, or posters/leaflets. The survey introduction makes it clear that responses will not be shared with the individual doula. In addition, The Open University researchers do not have direct access to bereaved participants, nor do they have their personal contact details. Everyone invited to the survey is informed that they can email the Primary Investigator to receive a copy of the report.

There is no financial remuneration for survey participants: we have not offered to pay or run a prize draw for completing the survey. This is because it is similar to bereavement surveys sent out by NHS Trusts/Office for National Statistics that do not offer payment/prize draws. At research conferences, this has been discussed, with some members of the public saying they would feel ‘wrong’ for accepting payment to provide feedback about someone’s quality of dying/death. By not offering payment, we are also minimising the volume of personal data being collected alongside survey responses.

It is intended that the survey will be ‘live’ during the period of the NHS-commissioned pilot and several months thereafter to enable the 3-month follow-up period. After that point, End of Life Doula UK will have access to the survey questions to use in paper format for future use (or to host on another survey platform) and will be the data controllers.

### Proposed analysis

The survey design is mixed methods. Of the 18 questions, 8 have five-point scales and 2 are free-text boxes; the remaining questions are multiple choice. For the quantitative data, the Jisc Online Survey platform provides visualisation tools to display quantitative data (table, bar and pie chart) and functionality for cross-tabulations. The qualitative data (responses to free-text questions) can be analysed using content analysis at a manifest level, to identify what has been said and group similar comments together (and where volume renders it meaningful to do, capture the frequency).^37^

The survey asks questions related to the support provided by the EoL doula to the respondent’s relative or friend and the support provided by the EoL doula to them and their relationship with the doula. Part 4 of the survey in particular asks about the respondent’s confidence and well-being before and after doula support. There are two questions in this section with a Likert five-point scale and are designed to enable a comparison between the before and after. There are four questions about the place of death (Part 5), which is one of the impact measures End of Life Doula UK had agreed with the commissioners and are to be analysed in conjunction with one another. The survey questions in this section aimed to understand where someone died, if the respondent knew the person’s preference, if the respondent felt the person died in their preferred place and how prepared the respondent felt for the person dying in the place that they did (even if it was not the preferred place of death). This combination of questions means that the analysis is less on equating home deaths with a good death,^38^ but understanding how doula support may have aided understanding preferences and preparedness. It is anticipated that analysis of the data will take place in late 2024.

## Reflection on the survey

The objective of this article is to outline the development of the survey tool. Nevertheless, when reflecting on the tool it is useful to consider the potential response rate and usefulness of data gathered. A survey design was in line with End of Life Doula UK’s aim to demonstrate the impact of doula support towards the EoL to healthcare commissioners, as well as to use the survey as a form of feedback to inform ongoing survey development. Relatively, low numbers of respondents would mean that any data reporting needs to be caveated about generalisability; however, others have found that nonresponse bias in EoL care feedback may not necessarily considerably shift results in terms of how services are rated.^39^ Moreover, as we have advocated with End of Life Doula UK, the best evaluative strategies also do not rely on single measurements to report on outcomes. Therefore, there is the ability to triangulate the survey responses with routine data collected via anonymised referral notes and client notes to support reporting against outcome measures.

The survey questions are publicly available, enabling others to use it in their evaluations or research with attribution. The tool itself has not been fully validated; following use in the pilot, there is scope to review its use, refine the tool and fully validate it. Until we have data, we are unable to comment on how successful the tool is in capturing the intended outcome measures or what comments participants may have about the tool. End of Life Doula UK intends to use the tool for ongoing work. With referral numbers increasing over the past year, and the fact that surveys are not being sent to the bereaved until at least 3 months after the bereavement, it is hoped that response rates will increase in line with the number of surveys sent out over the coming months. With the Leeds project continuing for an estimated 6 months at the time of writing, there is scope for a greater amount of data to be collected. There is also the potential for the tool to be used as part of the evaluation of a second End of Life Doula UK NHS commission in southwest London due to start at the beginning of 2024.

## Discussion

EoL doula support is a relatively new area of provision for dying people and those close to them, such as family and friends. The outline of the approach to the literature review undertaken prior to developing the bereavement survey shows the wide-ranging areas of possible impact of an EoL doula and the challenge of attempting to explore and outline these.

Arguably, it is even more innovative to have doula support commissioned as part of a locality’s healthcare service, such as reported here. Outcome measures that are meaningful within the EoL process for diverse individuals are not necessarily obvious. While published guidance appears to have been used as a starting point, emergent learning showed that the wider systems around the dying person and their contacts also need to be considered.

Much of what has been researched to date is from the perspectives of doulas.^9,11,15,40,41,42^ There is a dire need for empirical research on how doula support at the EoL is perceived and experienced and what potential impact it has on quality of life and death, bearing in mind that doulas support more than just the dying person. The need to do no harm with this research intervention complicated the information-gathering process. It might be that earlier active use of a version of the survey might yield valuable insights and observations in some cases where friends and family were around. Other narrative or creative methods may help the dying person and their family express something in a way that was potentially interesting to take part in rather than feeling like a customer satisfaction survey. Although the survey was quite different from those provided by NHS services, it might have come at a time when other such satisfaction surveys were being received.

Original scoping of existing survey tools provided a wealth of knowledge and tools regarding quality-of-life issues and symptom management yet in comparison there were limited tools to address and hear the voice of the bereaved. This could be for a number of reasons. Contacting the bereaved may feel like an intrusion on a person’s grief or asking them to revisit a period of time that they are trying to move on from may feel insensitive. For some services, the scope of their actual service finishes when the person they have been caring for dies. It was very evident from the literature that there were no tools to evaluate the role of the doula; thus, the challenge was to develop a survey that would not only evaluate the service but also capture the voice of the bereaved. The CODE tool provided a valuable starting point as it is a validated tool that seeks to capture data from both the dying patient and the family or carer.^30^ The themes from CODE correlated well with those from the National Study of Bereaved People (VOICES) survey,^43^ and by utilising the response options from CODE it conferred the developed post-bereavement survey some degree of face validity. The review of the tool by a previous EoL doula service user provided the research team with valuable insight into the appropriateness and usability of the tool and surrounding survey text.

Using surveys to capture experiences and ratings of services is relatively common practice in a wide range of settings, including in EoL care.^43–45^ For this project, it was deemed impractical and potentially inappropriate to systematically seek direct feedback from the dying persons being supported by the doula, especially in cases where doula support was only provided in the last days of life. Instead, we have opted to survey those close to them in line with other EoL care service evaluations that survey bereaved persons.^43,44^ Whilst some may use bereaved persons as a proxy, we have sought to design the survey to acknowledge that all responses are from the perspectives of the bereaved person and that they, too, were recipients of doula support.

## Strengths and limitations

The tool is designed to primarily be filled in online for optimised use and formatting of questions. Digital literacy may be an issue for some potential participants. Age UK estimates that nearly two million over 75 years are digitally excluded which may have been a barrier to participation for some.^46^ Online, participants must click ‘submit’ at the end of the survey to save their answers; thus, there is the risk that some participants unintentionally do not do this final step and their data are consequently unavailable. Paper versions of the survey are available but do not automatically auto-route where applicable and may appear long to potential participants due to font sizing that aids accessibility. For paper copies, participants may find it burdensome to return them via the post despite the inclusion of a stamped addressed envelope (i.e. they may not regularly send letters or not have easy access to postal services).

Another consideration is the timing of when the survey is actually sent. As a team, the decision was made to send the invite to complete the survey 3 months post-bereavement, although the doula who had been supporting the client and family had been asked to introduce the notion of the survey during their involvement with them. It was believed that this time would not be too close to the death nor so far in the future that the bereaved may find it difficult to recall. However, O’Connor suggests that this may be a difficult point in the bereavement process, at least in terms of the grieving brain and the difficulties with cognitive rational processes, which is what a survey may demand. Thus, going forward, it may be necessary to consider and review when the survey is introduced and sent.^47^

The strength of the approach adopted here is the ability for the survey to be designed bespoke for End of Life Doula UK, tailoring it both to their intended areas of influence with clients and to the outcome measures of the commission. This complements other data they are collecting but enables the collection of data directly from a person close to the dying person. Another strength of the survey tool is that it allows for the collection of data about both the dying person and the survey respondent, which means we are not conflating the two and assuming similar experiences between them. Like all bereavement surveys, one limitation is that it is not possible to collate retrospective user experience and feedback from the dying person.

## Conclusion

This paper has outlined the development of a survey that addresses the current dearth of tools that assess the quality of dying from the perspective of the bereaved and the fact that there was no existing tool for evaluating EoL doula support. The process of researching the area and designing the evaluation survey for this area of EoL care services was revealing of the complexity of the role and the difficulty of capturing what was found to be helpful for the dying person and those around them. The survey tool will continue to be trialled over the next year and further analysis may contribute to the understanding of the contribution and impact of EoL Doula in the United Kingdom.

## Supplemental Material

sj-docx-1-pcr-10.1177_26323524241273489 – Supplemental material for Understanding end-of-life doula care provision: reporting on the design of a bereavement survey to evaluate doula support Supplemental material, sj-docx-1-pcr-10.1177_26323524241273489 for Understanding end-of-life doula care provision: reporting on the design of a bereavement survey to evaluate doula support by Kirsten Bashir, Emma Clare, Catherine Pestano, Esther Ramsey-Jones and Erica Borgstrom in Palliative Care and Social Practice

## Declarations

**Future study:** This post-bereavement survey aims to hear the voice of the bereaved to help us understand, evaluate and improve this service. It is intended that further insights will be gained into the roles and scope of practice from the voices of the EoL doulas through a focus group and collaboratory workshop.

**Ethics approval and consent to participate:** Approval for this study was obtained following a full review from the Human Research Ethics Committee of The Open University with reference HREC/4400/Borgstrom on 12th July 2022. The consent to participate is not required or pertinent for this article.

**Consent for publication:** Not applicable.

**Availability of data and materials:** Data are currently not available. The survey developed for this study is available on ORDO at https://doi.org/10.21954/ou.rd.23735718.v1.
