# Supplementary material for: Understanding end-of-life doula care provision: reporting on the design of a bereavement survey to evaluate doula support
Source: Palliat Care Soc Pract. 2024 Oct 17;18:26323524241273489. doi: 10.1177/26323524241273489 (PMC11489920; doi:10.1177/26323524241273489)
Supplement: sj-docx-1-pcr-10.1177_26323524241273489 – Supplemental material for Understanding end-of-life doula care provision: reporting on the design of a bereavement survey to evaluate doula support [file sj-docx-1-pcr-10.1177_26323524241273489.docx]

**Questionnaire Design Checklist (adapted from Burns et al. 2008)**

**Title: Understanding End of Life Doula Care Provision: reporting on the design of a bereavement survey to evaluate doula support**

| **Section** | **Question** | **Yes** | **No** | **Comments** |
| --- | --- | --- | --- | --- |
| Abstract | Is the objective clearly stated? |  |  |  |
|  | Is the design of the study stated? |  |  |  |
|  | Is the study setting well described? |  |  | Paper is design only |
|  | Is the survey population described? |  |  |  |
|  | Are outcome measures identified? |  |  |  |
|  | Are the conclusions appropriate? |  |  | Doesn’t have a specific conclusion as not required by publishing journal |
| Introduction | Is the problem clearly stated? |  |  |  |
|  | Is the relevant literature cited and critically appraised? |  |  |  |
|  | Is the relevance of the research question explained? |  |  |  |
|  | Is the objective clearly stated? |  |  |  |
| Methods | Is the questionnaire design appropriate to the objective? |  |  |  |
|  | Is the setting clearly described? |  |  |  |
|  | Are the methods described clearly enough to permit other researchers to duplicate the study? |  |  |  |
|  | Is the survey sample likely to be representative of the population? |  |  |  |
|  | Is the questionnaire described adequately? |  |  |  |
|  | Have the validity and reliability of the questionnaire been established? |  |  | Type of validation discussed in the paper |
|  | Was the questionnaire administered in a satisfactory way? |  |  | Plans for administration discussed in the paper |
| Results | Does the questionnaire clearly address the objective? |  |  |  |
| Discussion | Is the questionnaire design succinctly summarised? |  |  |  |
|  | Are the implications of the questionnaire stated? |  |  |  |
|  | Are other interpretations considered and refuted? |  |  |  |
|  | Are the limitations of the study and its results explained? |  |  | Content limited to design rather than results as paper focused only on design |
|  | Are appropriate conclusions drawn? |  |  |  |
